# Exoscopic Visualization for Transorbital Surgery: Preliminary Anatomical and Clinical Validation Study

**DOI:** 10.3390/jcm14228165

**Published:** 2025-11-18

**Authors:** Francesco Corrivetti, Matteo de Notaris, Sergio Corvino, Amedeo Piazza, Edoardo Porto, Stefano Leo, Carlo Cavaliere, Matteo De Simone, Giorgio Iaconetta, Doo-Sik Kong

**Affiliations:** 1 Laboratory of Neuroanatomy, European Biomedical Research Institute of Salerno (EBRIS) Foundation, 84125 Salerno, Italy; matteodenotaris@gmail.com (M.d.N.); sergio.corvino@aslnapoli1centro.it (S.C.); amedeo.piazza@uniroma1.it (A.P.); edoardo.porto@istituto-besta.it (E.P.); s.leo@ebris.eu (S.L.); 2 Neurosurgery Unit, University Hospital “San Giovanni di Dio e Ruggi D’Aragona”, 84131 Salerno, Italy; matdesimone@unisa.it (M.D.S.); g.iaconetta@unisa.it (G.I.); 3 Department of Neurosurgery, Ospedale del Mare, ASL Napoli 1, 80147 Naples, Italy; 4 Department of Neurosurgery, Sapienza University, 00185 Rome, Italy; 5 Department of Neurosurgery, Fondazione IRCCS Istituto Neurologico Carlo Besta, 20133 Milan, Italy; 6 Department of Neurosurgery, Ospedale Santa Maria, 05100 Terni, Italy; 7 Department of Neuroradiology, IRCCS SDN, 80143 Naples, Italy; carlo.cavaliere@synlab.it; 8 Department of Neurosurgery, Samsung Medical Center, Sungkyunkwan University School of Medicine, Seoul 06351, Republic of Korea; neurokong@gmail.com

**Keywords:** exoscope, transorbital, neuroendoscopy, cadaveric dissection

## Abstract

**Background/Objectives:** The endoscopic transorbital approach (ETOA) is a minimally invasive surgical route that provides access to the lateral skull base through the superior eyelid. Originally developed as an endoscopic procedure, ETOA has recently been explored using alternative visualization tools such as the exoscope. This study evaluates the effectiveness of exoscopic visualization across the different steps of transorbital surgery. **Methods:** Eight formalin-fixed cadaveric specimens (16 sides) were dissected by four teams of neurosurgeons trained in ETOA. The dissection protocol consisted of three stages: skin, orbital, and intracranial. The teams were assigned to four groups: the first performed a pure endoscopic ETOA (group A) and the second and third performed a combined exoscopic/endoscopic ETOA, using exoscopic visualization, respectively, for the skin phase only (group B) or for the skin and orbital phases (group C), while the fourth group performed a pure exoscopic ETOA All surgeons rotated across groups. Operative time was recorded. After each procedure, surgeons rated operative comfort, maneuverability, and image quality on a 0–5 scale. Pre- and postoperative CT scans were used for volumetric analysis, comparing surgical cavity size with and without the endoscope in place. In addition, an illustrative exoscopic case was included. **Results:** Exoscopic visualization proved to be more effective during the skin phase. In the orbital phase, it improved access and reduced crowding during lateral wall drilling. However, endoscopic visualization provided superior image clarity and magnification for deep and medial orbital structures. CT-based analysis confirmed that the exoscope significantly improves the working space during orbital dissection. Moreover, the combined approaches (Groups B and C) achieved shorter operative times and higher subjective ratings. **Conclusions:** The exoscope could be a valuable visualizing tool for transorbital surgery. While the skin phase benefits most from exoscopic visualization, the endoscope remains essential for the intracranial phase. The orbital phase can be effectively performed with either technique, each offering specific advantages and limitations.

## 1. Introduction

The exoscope is a new hybrid visualization tool recently introduced in neurosurgical practice [1]. This optical instrument combines characteristics of both operative microscope and endoscope. It offers high resolution images and a wide viewing angle while remaining positioned outside the patient at a variable distance from the surgical field. The image is displayed on an external monitor, allowing heads-up visualization and improved ergonomics for the surgeon [2,3].

Similarly, the endoscopic transorbital approach (ETOA) is a novel minimally invasive route that provides access to the anterior and middle cranial fossae. Its introduction has expanded the neurosurgical armamentarium by offering a direct corridor to the skull base through the superior eyelid, with reduced manipulation of neurovascular structures [4,5,6].

Being a minimally invasive route, considering the narrow surgical corridor, ETOA was initially conceived as a pure endoscopic approach [7,8]. The rapid translation of ETOA from anatomical studies to the validation in clinical practice underlined its significant advantages leading to its widespread diffusion in neurosurgical practice [9,10,11,12,13,14,15,16,17]. As the indications for ETOA expanded to include a wide spectrum of skull base pathologies, surgical techniques have undergone progressive refinement. Some authors have described the combined use of exoscopic and endoscopic visualization to enhance different phases of the procedure [6,18]. Moreover, a recent paper, illustrates the utilization of the exoscope [19] as the sole and alternative visualization source to perform ETOA.

The aim of this anatomo-clinical study is to prove the effectiveness and feasibility of exoscopic visualization for transorbital surgery.

## 2. Materials and Methods

Anatomical dissections were performed at the Laboratory of Neuroscience of the EBRIS Foundation (European Biomedical Research Institute of Salerno, Italy). Eight adult formalin-fixed cadaveric specimens (16 sides), injected with red latex, were used.

The dissections utilized a 3D exoscope (VITOM 3D, Karl Storz SE & Co. KG, Tuttlingen, Germany), consisting of a high-definition camera mounted on a static holder arm. The video signal was processed through the Telepack+ system (Karl Storz SE & Co. KG, Tuttlingen, Germany). Illumination of the surgical field was provided via fiber optics connected to the exoscope’s camera and powered by a Power LED 300 light source (Karl Storz SE & Co. KG, Tuttlingen, Germany).

Endoscopic visualization was performed using a rigid 4 mm-diameter endoscope with a length of 18 cm, available in 0° and 30° rod lens configurations. All specimens underwent pre- and postoperative multislice helical computed tomography (CT) scans. Three-dimensional reconstructions from these scans enabled quantitative analysis of the transorbital approach, specifically comparing the surgical cavity volume with and without the endoscope present. This study was approved by the Local Ethics Committee (EBRIS/NEURO 2024-06). Cadaveric specimens were provided to our laboratory from MedCure, Inc. (Portland, OR, USA).

### 2.1. Stepwise Dissection

A standard superior eyelid ETOA was performed to provide transorbital access to the middle cranial fossa and exposure of the Meckel’s cave after interdural dissection of the cavernous sinus [20].

The approach was divided into three phases: (1) Skin Phase; (2) Orbital Phase; (3) Intracranial phase.

### 2.2. Dissection Protocol

The specimens were divided into four groups, each consisting of four specimens (eight sides), based on the visualization protocol used to perform the surgical approach:

- Group A: the endoscope was used as the sole visualization tool.
- Group B: ETOA was performed using exoscopic visualization for the skin phase and endoscopic visualization for the orbital and intracranial phases.
- Group C: exoscopic visualization was used for the skin and orbital phases, while the endoscope was used for the intracranial phase.
- Group D: ETOA was performed entirely under exoscopic visualization.

Four neurosurgeons (FC, SC, AP, EP), all trained in transorbital surgery, performed a standard ETOA on both sides of one head specimen per group (A to D), resulting in eight procedures using each visualization modality. Each procedure was timed by an independent evaluator, and the time data were recorded in a spreadsheet for statistical analysis. At the end of each procedure, a questionnaire was administered to the surgeon to evaluate, on a 0–5 scale: (1) the quality of visualization, (2) surgical comfort, and (3) surgical maneuverability. Scores ranged from 0 to 5, where 0 = poor, 1 = fair, 2 = adequate, 3 = good, 4 = very good, and 5 = excellent, according to the surgeon’s perception of visualization.

### 2.3. Statistical Analysis

All analyses were performed using R version 4.4.0 RC (16 April 2024) under Windows 11 × 64, with the readxl and rmarkdown packages. Normal distribution and homogeneity of variances were verified using the Shapiro–Wilk test and Bartlett test, respectively. A one-way ANOVA was then performed to compare operative times among the four groups (A–D). A *p*-value < 0.05 was considered statistically significant.

The data analyzed correspond to the operative timing measurements collected for each specimen which was generated directly from the R code. Questionnaire-based evaluations were analyzed descriptively, while no statistical comparison was performed for the volumetric measurements, which were used for geometric quantification only.

### 2.4. Skin Phase

The skin phase can be summarized in three steps

**Figure 1:**
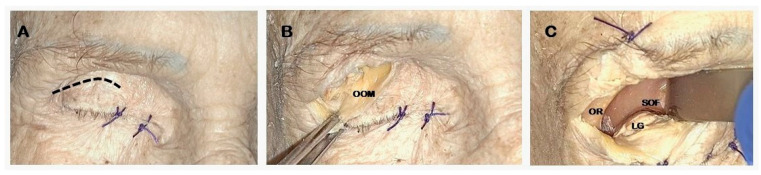
ETOA skin phase. Exoscopic visualization: (**A**): Superior eyelid skin incision; (**B**): subcutaneous dissection; (**C**): orbital access using a malleable retractor. LG: lacrimal gland; OOM: orbicularis oculi muscle; OR: orbital rim; SOF: superior orbital fissure.

1. Skin incision along the superior eyelid crease (Figure 1A).
2. Subcutaneous dissection of the orbicularis oculi muscle (OOM) to expose the orbital septum underneath (Figure 1B).
3. Lateral orbital rim exposure: the periosteum was cut acceding the intraorbital space, proceeding with the dissection in a sub-periosteal plane separating the periorbita from the lateral orbital wall and exposing the ventral surface of the greater sphenoid wing and the lateral part of the orbital roof. A malleable retractor was then inserted to displace the orbital content medially (Figure 1C).

### 2.5. Endo-Orbital Phase

**Figure 2:**
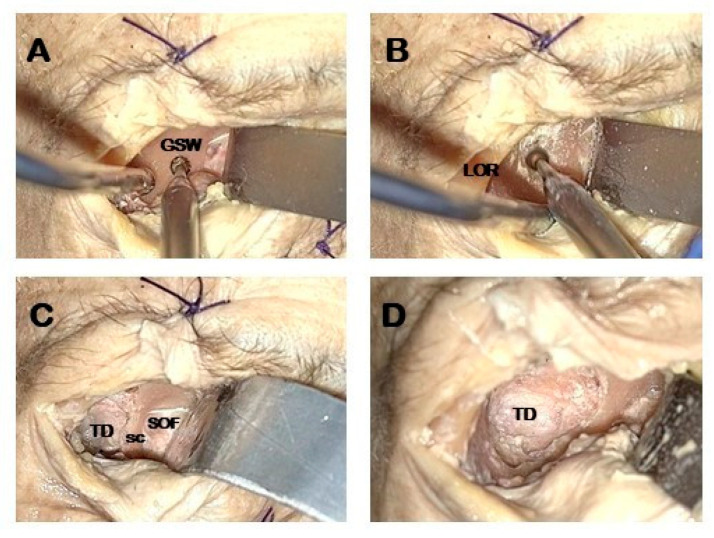
Orbital phase. Exoscopic visualization (**A**,**B**): exposure and drilling the ventral aspect of the greater sphenoid wing (GSW). (**C**,**D**): Exposure of the dura mater of the temporal pole and sagittal crest. GSW: greater sphenoid wing; LOR lateral orbital rim; SC: sagittal crest; SOF superior orbital fissure; TD temporal dura.

1. Lateral orbital wall exposure and orbital retraction: dissection is led in a subperiosteal plane detaching periorbita until the lateral edges of the superior and inferior orbital fissures are encountered.
2. Temporal fossa access: proximal portion of the lateral orbital wall of the greater sphenoid wing (GSW) is drilled acceding to the temporal fossa (Figure 2A).
3. GSW drilling: distal drilling of the lateral orbital wall along the GSW exposes the dura mater of the temporal pole (Figure 2B).
4. Sagittal crest (SC) resection: SC is a triangular bony ridge remnant of the GSW drilling dividing the medial temporal dura from the postero-lateral periorbital, that has to be removed to expose the entry point to perform interdural dissection [21] (Figure 2C). This maneuver allows the wide exposure of the dura mater of the temporal pole (Figure 2D).

### 2.6. Endocranial Phase and Meckel’s Cave Exposure

**Figure 3:**
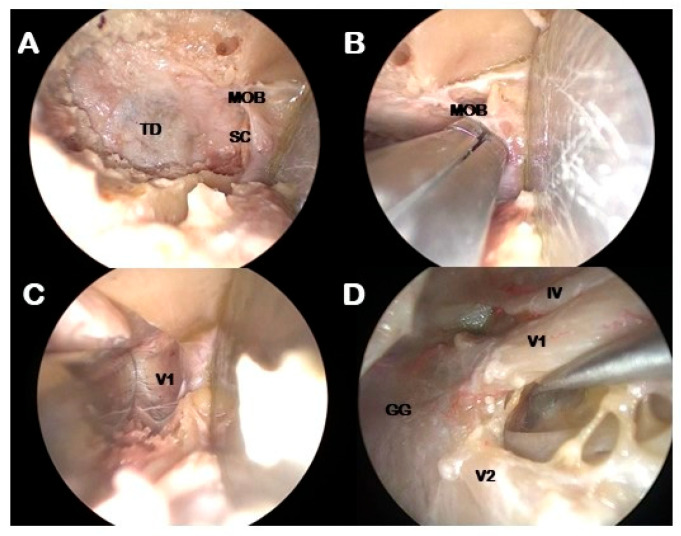
Intradural phase. Endoscopic visualization: (**A**): Exposure of the temporal dura. (**B**): Sagittal crest remotion and identification of the meningo-orbital band. (**C**): interdural dissection of the cavernous sinus. (**D**): exposure of the lateral wall of the cavernous sinus up to the Meckel’s Cave. GG: gasserian ganglion; IV: troclear nerve; MOB: meningo-orbital band; SC: sagittal crest; TD: temporal dura; V1: ophtalmic nerve; V2: maxillary nerve.

1. Middle fossa floor flattening: High speed drilling of the GSW is pursued inferiorly to flatten the middle temporal fossa floor (Figure 3A).
2. Meningo-orbital band (MOB) cut: MOB is a dural duplication that attaches the fronto-temporal dura with the periorbita with a horizontal orientation [22]. It must be cut in close proximity to the periorbita in order to free the dura propria from the posterior periorbita [20] (Figure 3B,C).
3. Interdural dissection of the CS: peeling the outer layer of dura propria from the inner dural layer constituted by the epineurium of the third, fourth and ophthalmic cranial nerves exposes the lateral wall of the CS up to the lateral wall of the Meckel’s cave [23] (Figure 3D).

### 2.7. Quantitative Radiological Analysis

3D reconstructions and volumetric measurements of the CT scans were performed using Materialise Mimics software (Materialise 3.2 version, NV, Leuven, Belgium). The endoscopic volume was calculated considering the average diameter of 4 mm of the endoscopic tip, while the average depth of the surgical field was estimated from the mean volume of the transorbital corridor among the eight specimens used for dissection. This approach allowed estimation of the space physically occupied by the endoscope tip during the orbital phase. No statistical comparison was performed, as the volumetric analysis was descriptive and intended to provide a geometric quantification of the available working space.

## 3. Results

Dissection was successfully completed in all groups. The results were independently evaluated using a 0–5 scale completed by each surgeon, assessing: perceived working area, surgical comfort, surgical maneuverability, and image quality (Table 1).

### 3.1. Skin Phase

Anatomical structures were clearly visualized with both devices. However, exoscopic visualization offered a significantly wider field of view and superior image quality in the external phase of the procedure. Its external optical system allows for a stable, panoramic visualization of superficial layers, improving depth perception and spatial orientation. In contrast, the endoscope, designed for use within enclosed cavities, provided a narrower and less defined image in this setting, reducing its effectiveness for dissection of superficial layers. As a result, the exoscope also improved surgical comfort and maneuverability during this phase.

### 3.2. Orbital Phase

During the orbital phase, the exoscope allowed excellent performance during lateral orbital wall dissection and drilling. This was mainly due to the use of only two instruments, which reduced crowding within the surgical corridor and improved maneuverability. In contrast, endoscopic visualization offered superior magnification and image clarity when dissecting and drilling in the medial portion of the surgical field, including sagittal crest removal and cutting of the meningo-orbital band.

Although both tools provided similar ratings in terms of surgical comfort, the exoscope’s ergonomic advantages may be underrepresented in subjective assessments. Moreover, the absence of the endoscope tip improved instrument freedom, as confirmed by volumetric CT analysis. However, the exoscope required frequent adjustments of the camera angle to maintain optimal visualization, especially during deeper steps such as GSW drilling.

### 3.3. Intracranial Phase

During the intracranial phase, endoscopic visualization provided clear advantages in terms of image quality and magnification, especially when dissecting the medial portion of the surgical field, such as during interdural dissection. The endoscope offered a more detailed view and better magnification when navigating the lateral wall of the cavernous sinus and Meckel’s cave.

The exoscope showed limitations in visual control within the deep and medial portions of the surgical field. The perceived working space appeared reduced due to its fixed viewing angle and the need for frequent repositioning to maintain proper alignment with the surgical target.

Overall, the endoscope proved to be more effective during the intracranial phase, particularly when dissecting medial anatomical structures.

Finally subjective evaluation data confirmed that groups using combined visualization (Groups B and C) obtained the highest mean scores for surgical comfort (4.1 and 4.3, respectively) and maneuverability (4.3 and 4.5), whereas the pure endoscopic group (Group A) showed lower comfort (3.2) and maneuverability (3.5). Image quality was rated highest for endoscopic visualization (4.6) and slightly lower for purely exoscopic control (3.9). These data indicate that hybrid visualization, especially when the exoscope was used during the skin and orbital phases (Group C), provided the most balanced combination of comfort, image quality, and operative efficiency.

### 3.4. Timing

**Figure 4:**
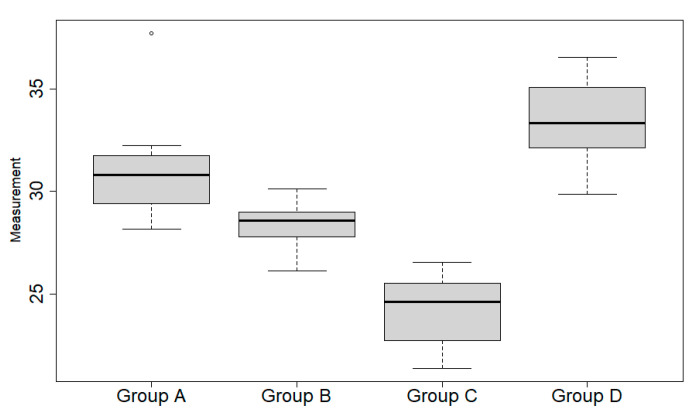
Boxplots correspond to time duration for each procedure/group. Each box is constituted by a horizontal wide black line, i.e., the median, and lower and upper horizontal thin lines, i.e., the first and third quartiles, respectively. The vertical lines, called “*whiskers*”, connect the box to the lowest and highest values within 1.5 times the interquartile range (i.e., the difference between third and first quartile). The single dot depicted at the top of group A boxplot is an *outlier*, because it falls outside the *whiskers* range.

All procedures showed significant differences regarding the duration of the procedure. The longest procedure was accomplished using the purely exoscopic approach. It is worth noting that both combined exoscopic–endoscopic controlled procedures (Groups B and C) showed a significant reduction in the duration of dissection when compared to the sole endoscopic (Group A) or exoscopic (Group D) procedures (Figure 4). Particularly, Group C exhibited the shortest mean dissection time (24.20 min).

#### Ergonomics

**Figure 5:**
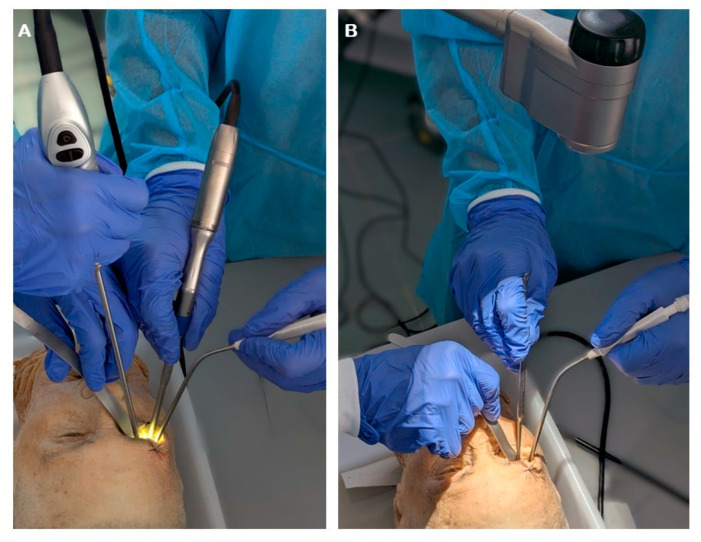
Operative setting using endoscopic (**A**) or exoscopic (**B**) visualization.

The ergonomic setting for transorbital surgery follows the same principles adopted for endonasal endoscopic procedures [24]. The patient is placed supine with the head turned approximately 15 degrees away from the side of the operation and secured in a Mayfield skull clamp, allowing for both stability and neuronavigation. The surgeon stands at the side of the patient, while the assistant is positioned just ahead, reproducing the configuration commonly used in endonasal approaches. This arrangement facilitates coordination between the two operators, enabling the assistant to manage the endoscope, suction, and additional instruments as required. In the endoscopic setting, however, the operative corridor is frequently crowded, as at least four instruments—including the endoscope tip and a spatula—occupy the narrow surgical space (Figure 5). By contrast, exoscopic visualization eliminates the presence of the endoscope tip within the corridor, thereby providing a significant gain in working space and improving instrument maneuverability. The main drawback of the exoscope is the need for frequent adjustments of the optical axis when advancing deeper into the orbit, which may intermittently interrupt the surgical workflow.

### 3.5. Quantitative CT-Based Analysis

3D reconstructions obtained from pre-and postoperative CT scans were used to quantitatively assess the surgical cavity. The segmentation process allowed differentiation between the total working volume and the space occupied by the endoscope tip during the at the beginning of procedure after the orbicularis oculi muscle dissection.

The 5 mm endoscopic tip was inserted at a mean length of 17.2 mm within the surgical cavity, allowing for precise volumetric calculation of the space physically occupied by the instrument.

The analysis demonstrated that the presence of the endoscope significantly reduces the free space available for maneuvering, in contrast, its absence resulted in a notable increase in residual working volume, potentially improving instrument handling and reducing crowding during the procedure.

**Figure 6:**
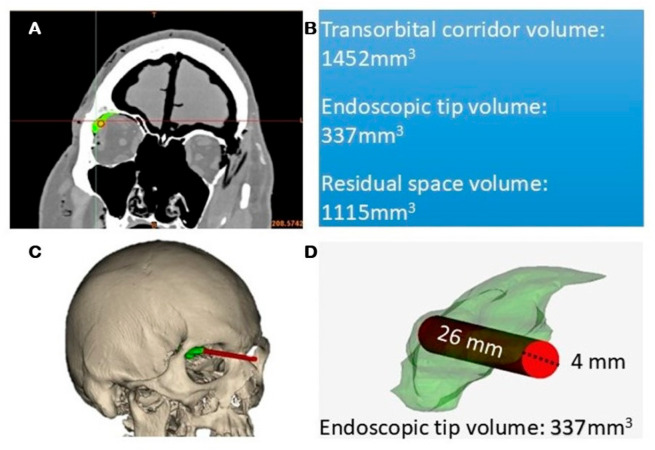
CT reconstruction of the transorbital corridor volume demonstrates the hindrance of the endoscopic tip during the orbital phase. (**A**): CT scan images, (**B**): quantitative analysis; (**C**): 3D scan reconstructions; (**D**): volumetric measurements.

This volumetric evaluation supports the benefit of incorporating exoscopic visualization, especially during the skin and orbital phases, by minimizing space occupation while maintaining adequate control and visualization (Figure 6).

### 3.6. Illustrative Cases

A 57-year-old male was referred to the neurosurgical department of the Samsung Medical Center in Seoul presenting intractable headache. Radiological examination was performed by means of brain magnetic resonance imaging (MRI) revealed a spheno-orbital meningioma of around 3 cm of maximal diameter. He underwent surgery using a HD 4K Vitom 3d exoscope (Karl StorzSE & Co. KG, Tuttlingen, Germany) and a rigid 4mm-diameter endoscope (Karl StorzSE & Co. KG, Tuttlingen, Germany).

**Figure 7:**
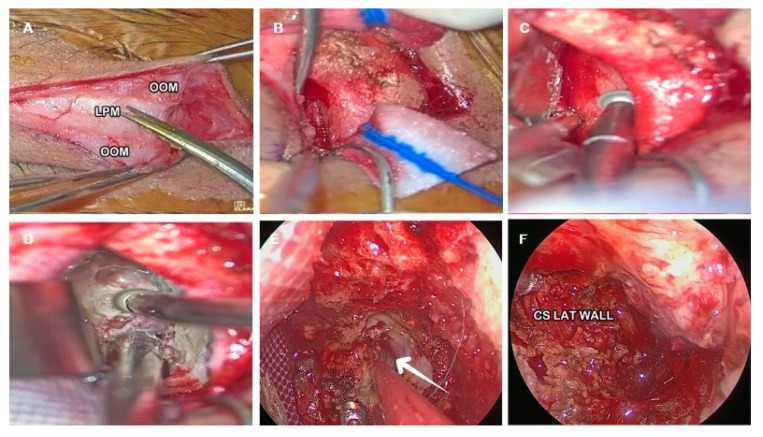
Illustrative case. (**A**): Skin Phase. (**B**): Subperiosteal dissection of the orbital rim and lateral orbital wall. (**C**): Drilling the proximal portion of the lateral orbital wall. (**D**): Drilling the ventral aspect of greater sphenoid wing exposing the dura mater of the temporal pole (white arrow). (**E**): Tumor dissection from the brain parenchyma. (**F**): Exposure of the lateral orbital wall after gross total resection. LPM: levator palpabrae muscle; OOM: orbicularis oculi muscle.

Exoscopic visualization was used to perform skin phase (Figure 7A). During orbital phase: subperiosteal dissection and exposure of the lateral orbital wall (Figure 7B) and proximal drilling of the lateral orbital wall to expose the temporal fossa was performed under exoscopic visualization (Figure 7C,D). The intracranial phase was performed under endoscopic visualization. First the lesion was detached from the brain parenchyma and then gross total resection was achieved through piecemeal resection (Figure 7E,F). Histological examination confirmed the suspected diagnosis of WHO grade I meningioma.

A postoperative MRI scan revealed complete resection. The patient was dismissed on day four without neurological deficits. The following illustrative clinical case is presented as an example of how exoscopic visualization can be applied in real surgical practice. It is intended to contextualize the anatomical findings rather than to validate them, as the study primarily focuses on anatomical and ergonomic evaluation).

## 4. Discussion

The exoscope is a visualization tool that has been recently introduced as an alternative to the operative microscope in neurosurgery. It provides a hybrid system that shares features with both endoscopic and microscopic visualization [25]. Similarly to the operative microscope, the exoscope is positioned outside the surgical field and uses a digital camera system to deliver light and magnification. However, it projects images on an external monitor, similar to the endoscope, offering advantages in terms of surgeon comfort, image control, and working distance [26].

Nonetheless, a main limitation of the exoscope, as well as of standard endoscopic systems, remains the lack of stereoscopic vision. However, this drawback has been partially addressed with the development of 3D visualization technology for both exoscopic and endoscopic systems, reducing the gap in stereopsis between the operative microscope and the exoscope.

Superior eyelid ETOA on the other hand, is a recently designed neurosurgical approach, being part of TONES [5], which includes four transorbital routes allowing minimally invasive access to the intracranial compartment. ETOA was initially conceived as a purely endoscopic approach, derived from the progressive refinements of endonasal techniques adapted to the transorbital corridor [27,28]. The progressive experience in transorbital surgery were able to determine its advantages and limitations, and, regarding the best setting for transorbital surgery, some authors, including our group, have reported improved surgical comfort using exoscopic view during the first steps of the approach [6,19].

In general, ETOA can be divided into three dissection phases. The first, or skin phase, allows access to the orbit through a superior eyelid incision. The second, or orbital phase, creates a corridor to reach the intracranial compartment, which constitutes the third phase of the dissection.

### 4.1. Skin Phase

Regarding the skin phase, endoscopic visualization, although feasible, appears to be particularly uncomfortable due to the limited illumination provided by the endoscope when used outside the anatomical cavities. As a result, the initial steps of ETOA, including the skin incision and subcutaneous dissection, are typically performed under direct vision or using surgical loupes.

However, setting up the operative microscope (OM) solely for the skin phase is often impractical, considering the short duration of this step compared to the time required to drape and position the microscope in and out of the field. For this reason, many surgeons have adopted the exoscope during the skin phase, achieving good results in terms of illumination, image quality, surgical comfort, and maneuverability. An additional benefit is the ability to begin the procedure using a screen-based external visualization system, which ensures visual continuity with the endoscopic phases that follow thus avoiding the transition from microscopic to endoscopic visualization [18].

### 4.2. Orbital Phase

Exoscopic visualization offers not only advantages during the skin phase but also plays a significant role during orbital dissection. The main goal of the skin phase is to create triangular access to the orbit, allowing enough space to introduce both the endoscope and its dedicated instruments. While the endoscope offers excellent visualization, the narrowness of the orbital corridor becomes a limiting factor as exposure progresses toward the intracranial compartment. In this setting, the presence of both the endoscopic tip and instruments within the confined space represents a significant hindrance.

The exoscope, on the other hand, allows the use of only two instruments inside the orbital corridor, thereby reducing crowding and improving maneuverability. It is also important to consider the quantitative findings obtained through CT-based volumetric analysis. The volume of the endoscopic tip within the surgical corridor, resulted in significative volume inside the orbital space. The subtraction of this volume from the total working cavity revealed a significant increase in residual maneuvering space. These data confirm that, beyond subjective perceptions of crowding, the physical presence of the endoscope considerably reduces the available surgical volume. However, the benefits of an external view are limited by the anatomical orientation of the orbital walls. The lateral orbital wall lies obliquely in the coronal plane, so the exoscope must be frequently repositioned and angled toward the orbital apex to adequately visualize the superior orbital fissure. Therefore, during the dissection of the most medial portion of the orbital space, for example, when performing sagittal crest removal, the endoscopic visualization proves to be more suitable. It is also worth noting that, in addition to the increased working space afforded by the absence of the endoscope, another practical advantage of the exoscope emerges during bone work. Drilling the greater sphenoid wing (GSW) under endoscopic control may provide a highly detailed view, but it often results in frequent lens contamination due to bone dust, requiring repeated interruptions for cleaning.

Therefore, the orbital phase seems to represent a transition point: while the exoscope is advantageous in the early steps, the endoscope becomes increasingly more effective as the dissection advances deeper into the orbit.

### 4.3. Intracranial Phase

The intracranial phase clearly highlighted the advantages of endoscopic visualization. As the surgical target extended deeper and more medially, the exoscope showed a progressive decline in image quality and illumination. The fixed external viewing angle made it difficult to maintain adequate visualization of medial structures, requiring frequent repositioning to align with the surgical target. These limitations reduced the perceived working space and affected the fluency of the dissection.

In contrast, the endoscope offered superior magnification and image clarity, particularly during dissection of the lateral wall of the cavernous sinus and Meckel’s cave. Its freehand maneuverability allowed for a more direct and continuous view of deep anatomical structures.

It is important to note that the present study focused exclusively on the standard ETOA, with the intracranial phase limited to the exposure of the lateral cavernous sinus and the floor of the middle cranial fossa. Although recent publications have described the extension of ETOA to regions such as temporal lobe [29], infratemporal fossa [30], sylvian fissure [31], and posterior fossa [9,11], these areas were not included in our analysis. Nonetheless, it is reasonable to assume that deeper surgical targets would further reinforce the advantages of endoscopic visualization (Table 2).

A brief comparison should also be made with the operative microscope, which remains the most widely available visualization tool in neurosurgical departments. While the exoscope and microscope share a similar external optical geometry, the exoscope offers a greater degree of freedom in positioning and a wider viewing angle, facilitating heads-up surgery and improved team ergonomics [32]. However, the ergonomic and visualization advantages observed with the exoscope are only partially transferable to the operative microscope, since the latter’s fixed ocular alignment and bulkier body may still limit flexibility in narrow working spaces such as the transorbital corridor. Therefore, although both systems provide external visualization, the exoscope appears more adaptable to the surgical geometry of ETOA.

### 4.4. Considerations

The choice of the optimal visualization tool for ETOA has to consider the specific requirements of each phase of the dissection. This study showed the exoscope is better suited for the initial stages while the endoscope remains superior in the intracranial phase due to its deeper magnification and image clarity.

A crucial difference between ETOA and endoscopic endonasal approaches (EEAs) lies in the nature of the surgical corridor. EEAs rely on a natural anatomical cavity that gradually expands, while ETOA requires the creation of a virtual space through dissection of the periorbita from the lateral orbital wall. As a result, the transorbital corridor is remarkably narrow in the orbital phase, progressively widening only after this step.

This anatomical constraint affects illumination patterns: endoscopes deliver a conic light beam optimized for natural cavities, whereas exoscopes and microscopes—offering external, reverse-cone illumination, are more effective in the early, confined stages of ETOA. These differences justify the improved comfort, maneuverability, and dissection time observed with combined exoscopic-endoscopic approaches.

Volumetric analysis further supports this hybrid strategy, highlighting how the physical presence of the endoscope can significantly reduce working space. These quantitative data complement the anatomical observations, bridging the gap between laboratory findings and their potential clinical implications. Phase-tailored visualization tools may therefore improve efficiency, ergonomics, and spatial management in transorbital surgery.

It is worth noting that ETOA, similarly to the traditional transcranial approaches, provides access to the paramedian compartment of anterior [33,34] and middle cranial fossae [35,36,37]. Indeed, its perspective over paramedian skull base compartments is similar to a transcranial corridor and therefore it benefit from the eternal visualization provided by the exoscope. Contrairly to transcranial approaches, ETOA provides an anteroposterior, retrobulbar axis of access toward the lateral skull base. Tailoring adequate working space during the orbital phase needs unavoidable degrees of orbital retraction. In this contex, exoscopic visualization reduces the total volume of instruments inside the orbital corridor and also provides less orbital compression reducing the risks of ophtalmological complications and improving the orbital outcome [38].

Beyond its technical benefits, exoscopic visualization may also offer significant educational advantages. The shared 3D monitor view enables all members of the surgical team, including trainees and multidisciplinary collaborators, to observe the same high-resolution image in real time. This feature promotes intraoperative teaching, enhances team coordination, and facilitates multidisciplinary discussion, which could further strengthen its integration into both training and hybrid neurosurgical settings.

### 4.5. Limitations

The use of cadaveric specimens presents important limitations. Tissue consistency, elasticity, and tolerance to retraction may differ considerably from those encountered in surgical conditions, potentially affecting the displacement of anatomical structures and the overall perception of maneuverability. Therefore, the anatomical observations reported may not be fully reproducible in clinical scenarios.

This study should be viewed as a complementary addition to the existing knowledge on visualization techniques in transorbital surgery. Its primary objective was to provide a structured comparison between exoscopic and endoscopic visualization tool for the different phases of the approach [39,40,41]. Further clinical studies are already planned to confirm these findings and evaluate their applicability in real surgical practice.

## 5. Conclusions

The exoscope appears to be a valuable tool in transorbital surgery. The initial skin phase is better performed under exoscopic visualization, offering improved ergonomics and a broader working area. In contrast, endoscopic visualization remains critical during the intracranial phase, where deep magnification and visualization around corners are essential.

As for the orbital phase, while the endoscope provides excellent visualization of deep anatomical structures, the exoscope offers several advantages, including reduced operative time and improved image quality, working space, surgical comfort, and maneuverability. These findings support a tailored choice of the visualization systems based on the phases of the surgical approach. Overall, these findings suggest that a phase-specific combination of exoscopic and endoscopic visualization may optimize transorbital procedures. Nevertheless, the present results should be interpreted with caution, as this is a preliminary anatomical and clinical validation study. Larger prospective clinical series are required to confirm these findings and to establish the precise role of the exoscope in skull base surgery.

## Figures and Tables

**Table 1:** Results of subjective evaluation of each visualization technique based on a 0–5 scale.

Group	Visualization Technique	Surgical Comfort	Maneuverability	Image Quality
A	Endoscopic only	3.2	3.5	4.6
B	Exoscopic (skin) + Endoscopic	4.1	4.3	4.4
C	Exoscopic (skin + orbit) + Endoscopic	4.3	4.5	4.2
D	Exoscopic only	3.6	3.8	3.9

**Table 2:** Advantages and limitations of exoscopic and endoscopic visualization for each ETOA phase.

EXOSCOPE		ENDOSCOPE	
Advantages	Limitations	Advantages	Limitations
Distance from the field allows improved instrument maneuverability Adjustable magnification Improved working space Avoid lens tarnishing during drilling	Requires constant adjustment to maintain proper angle No freehand control Poor visualization around corners Limited view in depth	Excellent visualization within orbital corridor High magnification and excellent deep-field visualization	Low resolution outside cavities Not designed for superficial layers Instrument crowding inside narrow corridor Lens tarnishing

## Data Availability

The data used in this study are unavailable due to privacy and ethical restriction.
